# Talc pleurodesis versus indwelling pleural catheter among patients with malignant pleural effusion: a meta-analysis of randomized controlled trials

**DOI:** 10.1186/s12957-020-01940-6

**Published:** 2020-07-23

**Authors:** Li Wang, Huan Deng, Xinling Chen, Can Li, Fengming Yi, Yiping Wei, Wenxiong Zhang

**Affiliations:** 1 Department of Thoracic Surgery, The Second Affiliated Hospital of Nanchang University, No. 1, Minde Rd, Nanchang, 330006 People’s Republic of China; 2 Department of Respiratory and Critical Medicine, The Second Affiliated Hospital of Nanchang University, Nanchang, 330006 China; 3 Jiangxi Medical College, Nanchang University, Nanchang, 330006 China; 4 Department of Oncology, The Second Affiliated Hospital of Nanchang University, Nanchang, 330006 China

**Keywords:** Talc pleurodesis, Indwelling pleural catheter, Malignant pleural effusion, Meta-analysis, Randomized controlled trials

## Abstract

**Background:**

Talc pleurodesis (TP) and indwelling pleural catheter (IPC) are used for the management of malignant pleural effusion (MPE). Our meta-analysis was conducted to assess the efficacy and safety of both treatments among patients with MPE.

**Methods:**

We acquired pertinent randomized controlled trials (RCTs) by searching PubMed, ScienceDirect, the Cochrane Library, Scopus, Ovid Medline, Embase, Web of Science, and Google Scholar. The endpoints included survival, pleurodesis rates, total drainage, further pleural interventions, hospital days, symptoms, quality of life (QoL), and complications.

**Results:**

We included four high-quality RCTs. Both treatments were effective among patients with MPE and no previous pleurodesis, with comparable survival and equivalent relief of breathlessness. Additionally, the TP group had higher pleurodesis rates, less total drainage, and fewer all-grade complications (including catheter blockage and cellulitis). However, patients in the TP group had more pleural procedures and relatively longer hospital stays. Additionally, no apparent difference was detected in QoL.

**Conclusions:**

TP has better pleurodesis rates, less total drainage, and fewer all-grade complications. However, TP has more pleural procedures and is not feasible for patients with trapped lungs. IPC has fewer further pleural interventions and shorter hospital stays. However, IPC has the nuisance of long-term in situ draining.

## Introduction

Malignant pleural effusion (MPE) affects over 750,000 patients every year across America and Europe, and its occurrence is increasing [1, 2]. MPE indicates the development of advanced stages of cancer among tumor patients, with a short mean life expectancy of approximately 4 months [3, 4]. MPE can lead to related symptoms, including dyspnea and chest pain [5, 6]. Inevitably, these clinical symptoms impair the quality of life (QoL) of patients who have MPE [7, 8]. Because the malignancy is terminal by the time MPE develops, the main purpose of treatment is symptom relief [9, 10].

For many years, pleurodesis has focused on the use of chemical agents (e.g., cisplatin, talc, and interleukin-2) [11–13]. In a recent network meta-analysis, Dipper et al. suggested that talc might have the best clinical efficacy for chemical pleurodesis [14]. Talc pleurodesis (TP) usually requires lengthy inpatient hospitalization during patients’ remaining days, which is a major disadvantage [15, 16]. As an alternative form of fluid management, indwelling pleural catheter (IPC) placement is an outpatient procedure that has been suggested to be as effective as traditional methods in relieving symptoms in MPE patients [17, 18]. However, IPCs cannot achieve the same pleurodesis rates as TP, although some recent studies report that spontaneous pleurodesis rates in IPC recipients vary from 16% to 65% among patients with MPE [19, 20]. Additionally, some clinical trials have reported inconsistent results regarding symptom remission. In a prospective RCT, Demmy et al. suggested that the IPC group had better relief of unilateral MPE than the TP group, especially among participants with trapped lungs [20]. However, several recent RCTs found no significant difference between the IPC and TP groups in palliative patient reports of dyspnea among participants with MPE and no previous pleurodesis [21–23].

Although a similar meta-analysis was published in 2019 [24], that analysis did not merge some important endpoints (visual analog scale (VAS) dyspnea scores) and ignored key endpoints (pleurodesis rates). Furthermore, the chemical pleurodesis group in that meta-analysis included two different chemicals (talc and doxycycline), and a prospective RCT [25] suggests that talc and doxycycline have different degrees of efficacy in the management of MPE, revealing the potential for large bias in that analysis. Our analysis makes several improvements compared with that analysis. (1) We compared only the TP and IPC groups, potentially increasing the accuracy and decreased the bias of our study compared to the previous meta-analysis. (2) Our study performs a quantitative synthesis of pleurodesis rates and VAS dyspnea scores, both of which are very important endpoints for assessing the efficacy of MPE management. (3) Our study provides registration information. Therefore, our study can offer the latest and most comprehensive evidence-based suggestions concerning the relative efficacy and safety of TP and IPC.

## Materials and methods

Our meta-analysis and written according to the PRISMA (Preferred Reporting Items for Systematic Reviews and Meta-Analyses) guidelines (Table S1) and registered in PROSPERO (registration information: PROSPERO CRD42019147776).

### Search strategy

Pertinent articles were obtained on February 2, 2020, through the following databases: PubMed, ScienceDirect, the Cochrane Library, Scopus, Web of Science, Embase, Ovid Medline, and Google Scholar. We searched the following terms: “talc pleurodesis”, “indwelling pleural catheter”, and “malignant pleural effusion”. Table S2 shows our detailed search strategy. The references of the included articles were searched for eligible studies not found in the initial search.

### Selection criteria

We included studies conforming to the criteria of the PICOS model (participants, interventions, comparisons, outcomes, and study design): (1) participants: patients with MPE have not undergone IPC or pleurodesis treatment previously; (2) interventions and comparisons: TP group vs. IPC group; (3) outcomes: survival, pleurodesis rates (defined in the TP group as no need for further pleural interventions (regardless of radiology), and defined in the IPC group as no obvious recurrence according to thoracic ultrasound or chest radiograph and no need for further pleural interventions after IPC removal following the spontaneous end of drainage [26]), total volume of drainage, symptoms, further pleural interventions (conducted for further fluid evacuation), QoL, total hospital stay, and adverse events (AEs); and (4) study design: RCTs written in English.

Observational studies, reviews, meta-analyses, conference papers, case reports, animal trials, and articles with the same patient sources were excluded.

### Data extraction

Two investigators (Li Wang and Huan Deng) independently extracted the following data: first author, publication date, source of participants, number of participants, participants’ traits (age, sex, tumor types, previous treatments), survival, pleurodesis rates, total volume of drainage, symptoms, QoL, further pleural interventions, total days of hospitalization, and AEs (all AEs and serious AEs). All disagreements were resolved by the third investigator (Wenxiong Zhang).

### Quality evaluation

The quality of all included trials was assessed using the new version (version 2) of the Cochrane risk-of-bias tool, which includes 5 major domains of risk: the randomization process, deviations from the intended interventions, missing outcome data, measurement of the outcome, and selection of the reported results [27].

We used the GRADE (Grading of Recommendations Assessment, Development and Evaluation) system to assess the therapeutic strategy and study the design of outcomes (survival, pleurodesis rates, symptoms, further pleural procedures, QoL, and AEs). GRADE contains a total of four grades (high, medium, low, and very low) [28].

### Statistical analysis

Our meta-analysis was conducted using RevMan (version 5.2) and STATA (version 12.0). Risk ratios (RRs) and 95% CIs were used to analyze pleurodesis rates (RR > 1 favors TP arm; RR < 1 favors IPC arm), further pleural interventions, mortality, and AEs (RR > 1 favors IPC arm; RR < 1 favors TP arm). Weighted mean differences (WMDs) with 95% CIs were used to analyze symptoms (WMD > 0 favors TP arm; WMD < 0 favors IPC arm). Standardized mean differences (SMD) with 95% CIs were used to analyze QoL (SMD > 0 favors TP arm; WMD < 0 favors IPC arm). Heterogeneity was assessed using the *χ*^2^ test and *I*^*2*^ statistic. When *I*^*2*^ > 50% or *P* < 0.10, showing obvious heterogeneity, we applied a random-effects model; otherwise, we applied a fixed-effects model. Publication bias was evaluated using Begg’s test and Egger’s test. *P* < 0.05 was considered statistically significant.

## Results

### Search results and quality evaluation

**Fig. 1:**
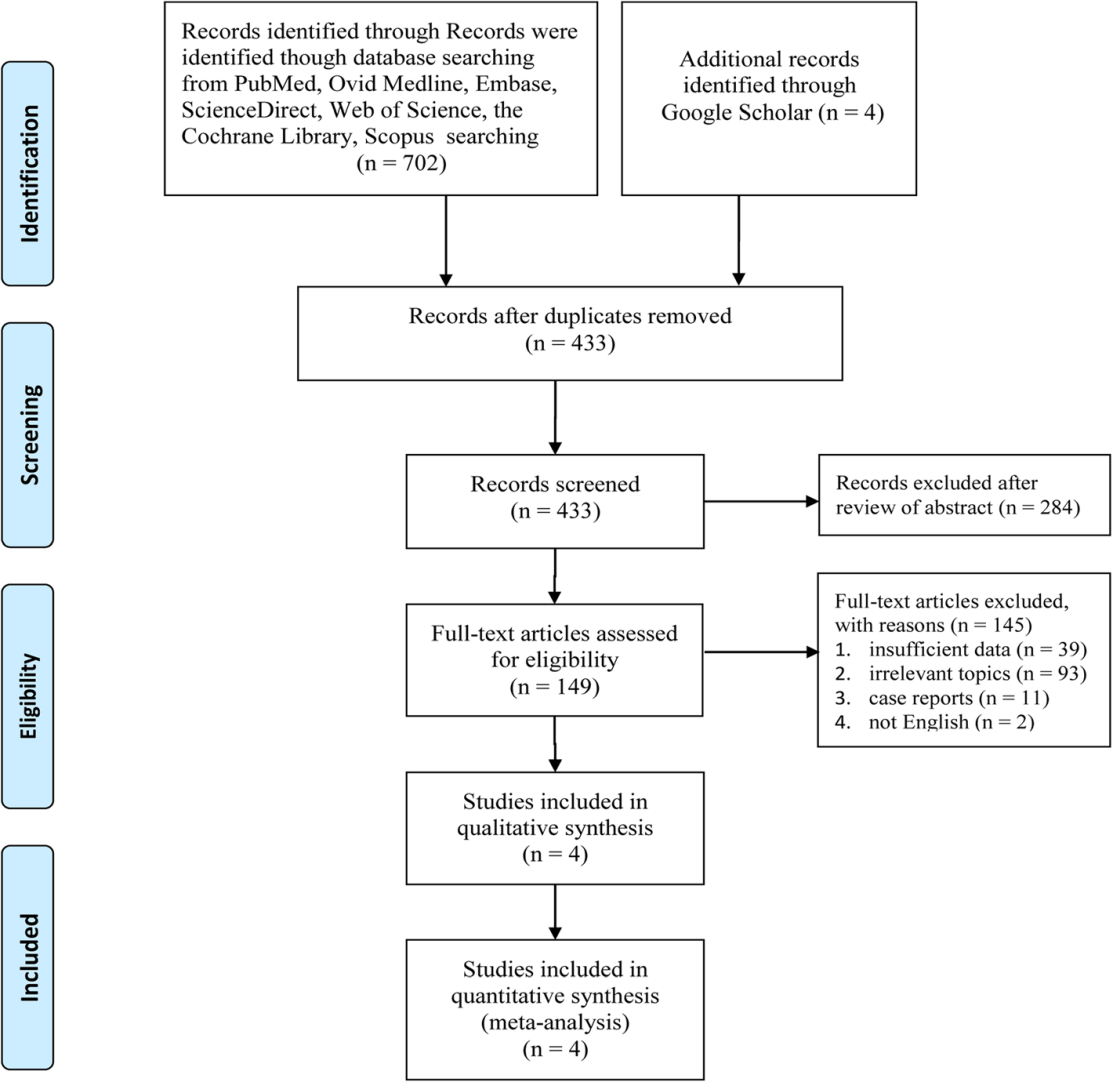
Flow chart of study selection

Figure 1 documents the study selection process in detail. Four RCTs including a total of 403 participants (TP, 203; IPC, 200) were eventually identified in our quantitative synthesis [20–23]. Table S3 clearly shows that most included studies had a low to medium risk of bias. In addition, Table S4 shows that most of our outcomes are high or medium according to the GRADE system, while others are low. Table 1 documents the baseline characteristics and major evaluation indicators of the included clinical trials.

**Table 1:** Characteristics of all included studies

Study		Nation	Groups	Patients (*n*)	Median age (year)	Sex		Tumor types (*n*)			TP group	Follow-up (month)	Design	
						Male	Female	Lung cancer	Breast cancer	Others	The installments of chest tube	Dose of talc		
2012	Demmy [20]	USA	TP	29	60	16	13	17	4	8	Chest catheter (≥ 24F)	4–5 g	NA	RCT
			IPC	28	64	17	11	19	3	6				
2012	Davies [21]	UK	TP	54	67	23	31	16	11	27	Chest tube (12F)	4 g	12	RCT
			IPC	52	67	23	29	9	16	27				
2017	Boshuizen [22]	Netherlands	TP	48	60	27	21	16	10	22	Chest tube (size: 15-20Ch)	NA	193^a^	RCT
			IPC	46	64	19	27	15	10	21				
2017	Thomas [23]	Australia	TP	72	70.5	42	30	29	4	39	Tube thoracostomy (12-18F)	Routine dose	12	RCT
			IPC	74	71	39	35	19	14	41				

*TP* talc pleurodesis; *IPC* indwelling pleural catheter; *mo* month; *F* French; *RCT* randomized controlled trail; *NA* not available

^a^The time of follow-up is calculated by day rather than month

### Survival

We evaluated the survival of patients in the TP and IPC groups in terms of 3-month mortality, 6-month mortality, and 12-month mortality.

Two RCTs [20, 21] reported 3-month mortality (heterogeneity: *I*^*2*^ = 66%, *P* = 0.09). Obvious differences were not detected between the TP and IPC groups (RR = 0.80, 95% CI: 0.29-2.16, *P* = 0.66; Figure S1A).

Only one trial [21] reported 6-month mortality, and there was no apparent difference between the groups (TP: 25/54; IPC: 27/52, *P* = 0.56).

Additionally, two trials [21, 23] reported 12-month mortality (heterogeneity: *I*^*2*^ = 81%, *P* = 0.02). No apparent difference was detected between the two groups (RR = 1.03, 95% CI: 0.76-1.40, *P* = 0.83; Figure S1B).

### Pleurodesis rates

**Fig. 2:**
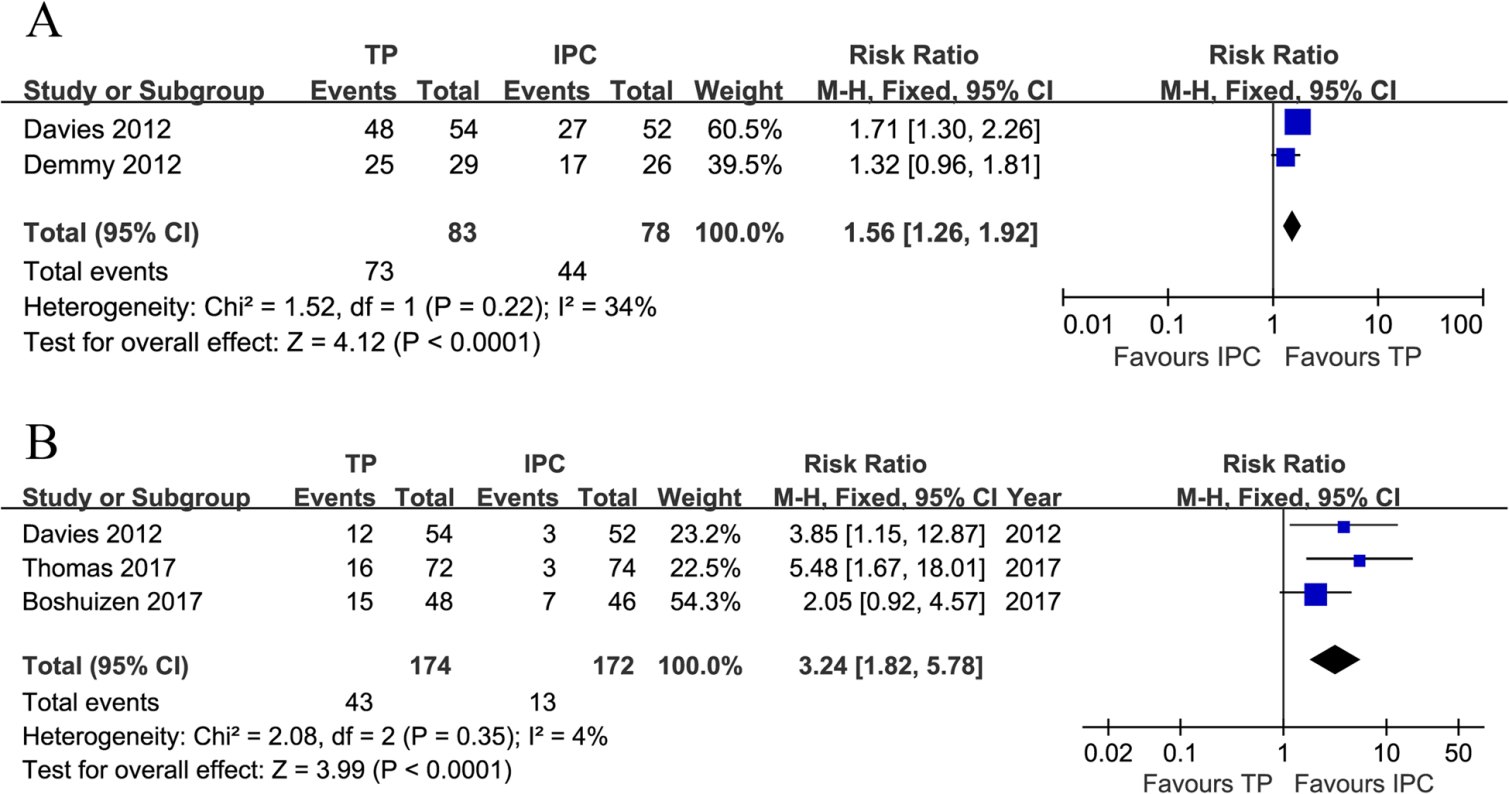
Forest plots of pleurodesis rates (**a**) and further pleural interventions (**b**) associated with TP versus IPC

Two trials [20, 21] reported pleurodesis rates (heterogeneity: *I*^*2*^ = 34%, *P* = 0.22). Pleurodesis rates were markedly higher in the TP group than in the IPC group (87.95% vs. 56.41%, RR = 1.56, 95% CI: 1.26-1.92, *P* < 0.0001; Fig. 2a).

### Total volume of drainage

Although only one trial [20] reported the mean and standard deviation (SD) of the total volume of drainage, the mean total drainage was considerably larger in the IPC group than in the TP group (IPC: 5802 ml; TP: 1911 ml; *P* = 0.04).

### Further pleural interventions

Three trials [21–23] reported further pleural interventions during the follow-up (heterogeneity: *I*^*2*^ = 4%, *P* = 0.35). The IPC group had fewer further pleural interventions than the TP group (RR = 3.24, 95% CI: 1.82-5.78, *P* < 0.0001; Fig. 2b).

### Total hospital stay

Only one trial [23] reported the mean and SD of total hospitalization time. The mean total hospital stay was longer in the TP group than in the IPC group (TP: 16.3 days; IPC: 12.7 days).

### Symptoms

We evaluated the symptoms of patients in both treatment groups with respect to VAS dyspnea scores and VAS chest pain.

Two trials [21, 23] compared VAS dyspnea scores at baseline (heterogeneity: *I*^*2*^ = 47%, *P* = 0.17) and VAS dyspnea scores after treatments (heterogeneity: *I*^*2*^ = 81%, *P* = 0.02). The two trials, especially that of Davies et al. [21], did not have perfectly matching baseline VAS dyspnea scores for both treatments (WMD = −2.32 mm, 95% CI: −8.59 to 3.94 mm, *P* = 0.47; Figure S2A). No significant difference was detected in VAS dyspnea scores after the treatments (WMD = 0.93 mm, 95% CI: −1.79 to 3.65 mm, *P* = 0.50; Figure S2B).

Only one trial reported VAS chest pain [21]. Although the mean and SD of scores were not shown, there was no apparent difference in the reduction in VAS chest pain between the two treatments (TP: 4.4 mm, 95% CI: 3.8-12.6 mm; IPC: 8.2 mm, 95% CI: 0.3-16.2 mm).

### Quality of life

Although two included studies [21, 23] reported the mean and SD of QoL scores, they adopted two different measures for evaluation (QLQ-30 and EuroQol 5-Dimension (EQ5D) QoL). Therefore, we used SMD to perform a quantitative synthesis of QoL, and no obvious difference was detected in this variable (WMD = −1.50, 95% CI: −3.80 to 0.80, *P* = 0.20; Figure S2C).

### Adverse events

The complications of both groups were compared in terms of all-grade AEs and serious AEs. Furthermore, we conducted subgroup analyses of the five most common toxicity events.

**Fig. 3:**
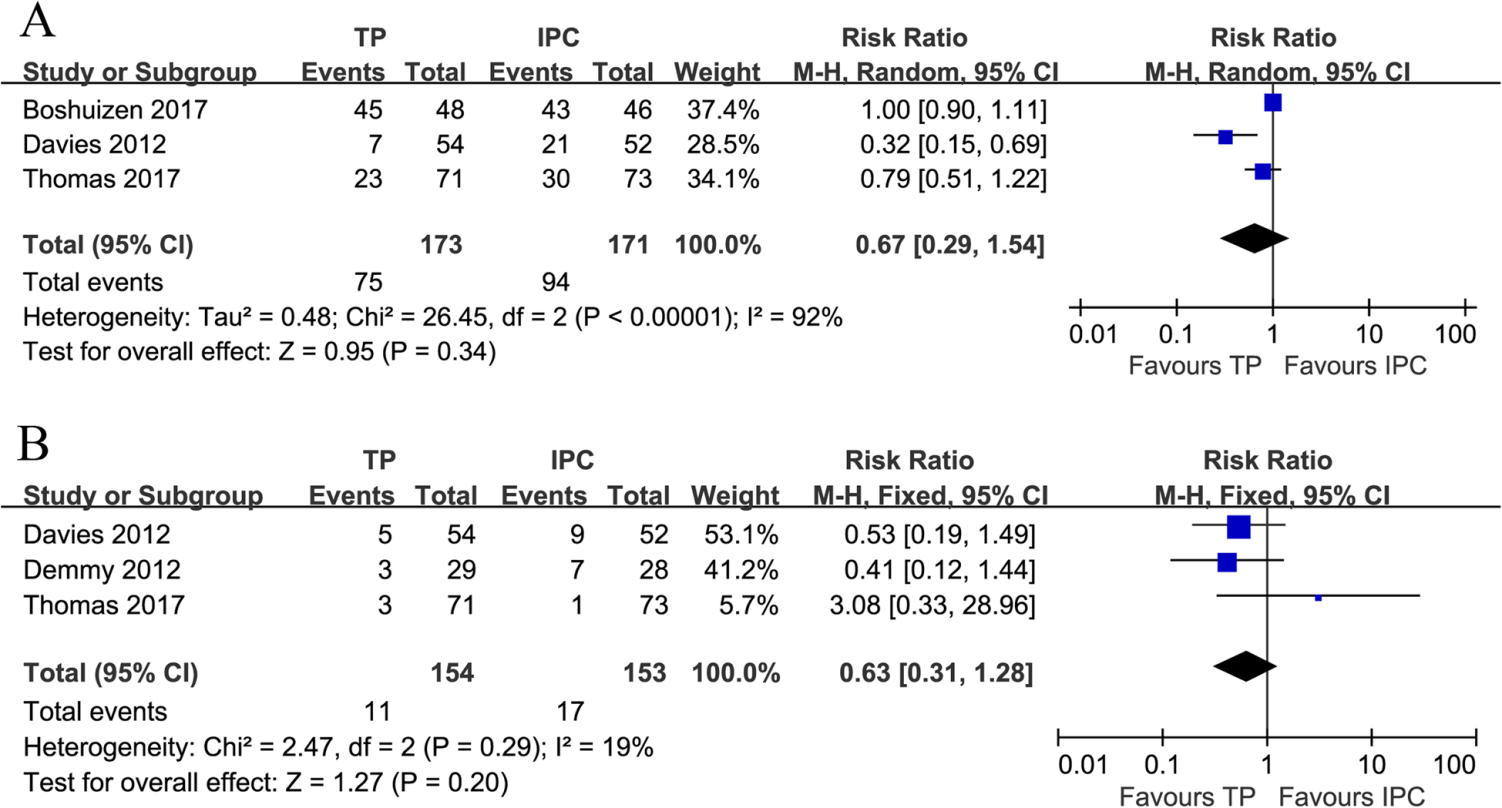
Forest plots of all AEs (**a**) and serious AEs (**b**) associated with TP versus IPC

Three trials [21–23] compared all AEs (heterogeneity: *I*^*2*^ = 92%, *P* < 0.00001). No obvious difference was found between the two groups (RR = 0.67, 95% CI: 0.29-1.54, *P* = 0.34; Fig. 3a).

Three trials [20, 21, 23] compared serious AEs (heterogeneity: *I*^*2*^ = 19%, *P* = 0.29), and no apparent difference was detected between the groups (RR = 0.63, 95% CI: 0.31-1.28, *P* = 0.20; Fig. 3b).

In the subgroup analysis of the five most common AEs (in order of incidence, dyspnea/breathlessness, catheter blockage, cellulitis, pleural infection, and pain), pooled results of all-grade AEs demonstrated no apparent intergroup differences in the incidence of dyspnea/breathlessness, pleural infection, or pain. Among all AEs, Table 2 indicates that the TP group had a lower incidence of catheter blockage (RR = 0.11, 95% CI: 0.02-0.57, *P* = 0.009) and cellulitis (RR = 0.14, 95% CI: 0.03-0.77, *P* = 0.02) than did the IPC group. Additionally, Table 3 suggests that no significant intergroup differences were found in any of the five serious AEs (in order of incidence: pleural infection, pain, dyspnea/breathlessness, catheter blockage, and cellulitis).

**Table 2:** Top five adverse events (all) associated with TP versus IPC

Adverse effects	No. of studies	TP group (event/total)	IPC group (event/total)	RR (95% CI)	*P* value	Heterogeneity	
						*I*^*2*^ (%)	*P* value
Dyspnea/breathlessness	2	8/119	7/119	1.15 [0.43, 3.06]	0.78	0	0.42
Catheter blockage	2	1/126	13/126	0.11 [0.02, 0.57]	0.009	0	0.82
Cellulitis	2	1/126	10/126	0.14 [0.03, 0.77]	0.02	0	0.85
Pleural infection	1	1/54	7/52	0.14 [0.02, 1.08]	0.06	NA	NA
Pain	1	1/48	2/46	0.48 [0.04, 5.11]	0.54	NA	NA

*IPC* indwelling pleural catheter; *TP* talc pleurodesis; *RR* risk ratio; *CI* confidence interval; *NA* not available

**Table 3:** Top five adverse events (serious) associated with TP versus IPC

Adverse effects	No. of studies	TP group (event/total)	IPC group (event/total)	RR (95% CI)	*P* value	Heterogeneity	
						*I*^*2*^ (%)	*P* value
Pleural infection	2	2/126	7/126	0.28 [0.06, 1.32]	0.11	0	0.54
Pain	2	1/100	3/101	0.61 [0.03, 12.92]	0.75	50	0.16
Dyspnea/breathlessness	1	2/29	0/28	4.83 [0.24, 96.42]	0.30	NA	NA
Catheter blockage	1	1/54	1/52	0.96 [0.06, 15.00]	0.98	NA	NA
Cellulitis	1	0/54	1/52	0.32 [0.01, 7.71]	0.48	NA	NA

*IPC* indwelling pleural catheter; *TP* talc pleurodesis; *RR* risk ratio; *CI* confidence interval; *NA* not available

### Subgroup analysis

Due to insufficient data in the trials included in our meta-analysis, we did not conduct a subgroup analysis of key outcomes. If new studies are published, a subgroup analysis of important results may be conducted.

### Sensitivity analysis

Both all-grade AEs (Figure S3A) and serious AEs (Figure S3B) clearly manifested robust results; specifically, no estimated value was beyond the 95% CIs.

### Publication bias

We could not find any evidence of publication bias in the results for all-grade AEs (Begg’s test, *P* = 0.296, Egger’s test, *P* = 0.139; Figure S4A) or serious AEs (Begg’s test, *P* = 1.000; Egger’s test, *P* = 0.225; Figure S4B).

## Discussion

Two common and effective methods, namely, TP and IPC, are used to relieve symptoms among patients with MPE. However, there remains an open question as to which method can yield greater benefits. This meta-analysis compares the efficacy and safety of TP vs. IPC for patients with MPE and no previous pleurodesis. The pooled results of the four RCTs showed no apparent intergroup difference in survival or relief of breathlessness. Additionally, the TP group was associated with higher pleurodesis rates, a lower total volume of drainage, and fewer all-grade AEs (including catheter blockage and cellulitis) than the IPC group. However, the IPC group needed fewer additional pleural interventions than the TP group and had a shorter overall hospitalization time.

Although symptom remission is a major purpose of treatments for MPE, survival is an important criterion of efficacy of both groups. Pooled outcomes showed no significant difference in 3-month mortality or 12-month mortality. A retrospective review also detected no apparent difference in postprocedural mortality (IPC: 4.35% vs. TP: 1.64%, *P* = 0.372) [29]. Analogously, a retrospective analysis demonstrated that participants in the thoracoscopic talc group had similar in-hospital mortality (8% vs. 3%, *P* = 0.41) to those treated with tunneled pleural catheters [30]. Moreover, a recent meta-analysis also showed no apparent difference in 3-month mortality between IPC and chemical pleurodesis (RR = 1.25, 95% CI: 0.45-3.45, *P* = 0.67) [24]. Thus, we can conclude that among patients with MPE, the survival of the TP group is equivalent to that of the IPC group.

The impact of pleurodesis is a crucial consideration in the efficacy of either procedure. Our outcomes suggest that the TP group had more successful pleurodesis than the IPC group. An RCT of 18 centers reported that the administration of talc through an IPC achieved higher rates of pleurodesis than an IPC alone (43% vs. 23%, *P* = 0.0008) as of day 35, and the talc group still had superior pleurodesis rates (51% vs. 27%, *P* = 0.003) on day 70 [26]. Successful pleurodesis promotes the adhesion of the parietal pleura and visceral pleura, and the production of MPE is greatly reduced [31]. Thus, owing to higher rates of pleurodesis, the TP group is expected to have a lower total volume of drainage than the IPC group. Undeniably, a lower total volume of drainage means a lower loss of protein, which is helpful in reducing patients’ need for protein supplementation. Moreover, our pooled outcomes suggest no apparent differences in symptom relief. Regarding the discrepancies in pleurodesis rates and VAS dyspnea scores after treatments, we arrived at a possible explanation. Specifically, the two included RCTs reporting VAS dyspnea scores did not perfectly match baseline VAS dyspnea scores between the two treatments. Although the baseline difference was not very obvious, the TP group had lower baseline VAS dyspnea scores than the IPC group, especially in Davies et al. [21], which might weaken the improvement of dyspnea in the TP group. Additionally, the VAS dyspnea scores of the TP group increased (*P* = 0.02) after the removal of Davies et al. [21], which further increases the reliability of our assumption. Therefore, TP might achieve better improvement of dyspnea than IPC according to our assumption. Our conclusions should be applied with caution, especially this assumption, and additional high-quality RCTs with large samples are still needed.

The influences of further pleural interventions and overall hospitalization days are also indispensable for appraising the efficacy of both treatments. Our results suggest that the IPC group had fewer further pleural procedures than the TP group, which seems to indicate better control of MPE. In a multicenter study, Fysh et al. also suggested that a lower proportion of participants in the IPC group than in the TP group needed further pleural interventions (13.5% vs. 32.3%) [32]. Furthermore, Hunt et al. reported that participants with tunneled pleural catheters had fewer ipsilateral reinterventions due to recurrent ipsilateral effusions than those using thoracoscopic talc (2% vs. 16%, *P* = 0.01) [30]. It seems inconsistent for the TP group to have both higher pleurodesis rates and more additional pleural procedures than the IPC group; we arrived at the following possible explanation. Although there is a reduced chance of pleurodesis in the IPC group, this does not cause a serious problem because the IPC can continue draining the reaccumulating effusion. However, in the TP group (for whom long-term drainage catheters are not inserted), this will result in a need for further pleural procedures to drain the fluid if pleurodesis fails (even if it fails at a lower rate than in the IPC group). In addition, for patients with recurrence after initial successful pleurodesis in the TP group, further pleural procedures must be performed to drain the reaccumulating pleural effusion. In a recent retrospective study, Liou et al. found that the total length of hospitalization was longer in the TP group than in the IPC group (11.1 vs. 9.7 days, *P* = 0.34) [33]. TP recipients were more likely than IPC recipients to require further pleural interventions and were hospitalized longer, which may lead to higher patient costs, but pleurodesis rates and the total volume of drainage are also very important for patients with MPE.

Examining complications plays an essential role in comparing the safety of the two treatments among patients with MPE. Our findings show that the TP group had a similar number of serious AEs to the ICP group but had a lower incidence of all-grade AEs, including catheter blockage and cellulitis. In an open-label RCT, Davies et al. suggested that patients in the TP group had fewer all-grade AEs (13% vs. 40%, *P* = 0.002), and no apparent difference was found in serious AEs between the two groups (odds ratio (OR) = 2.10, 95% CI: 0.57 to 7.71, *P* = 0.26) [21]. Analogously, Thomas et al. showed that the TP group had fewer patients who were affected by AEs of any grade (18% vs. 30%) [23]. It is no exaggeration to state that fewer all-grade AEs occur in the TP group than in the ICP group, than in the ICP group, which is beneficial for reducing unnecessary discomfort in their remaining life. Furthermore, the presence of fewer complications in the TP group might reduce the number of readmissions and even improve the QoL among advanced cancer patients with MPE.

Our study has several latent limitations. First, although all included articles are RCTs, the limited number of included trials (only 4 RCTs) may intervene with the quality of the outcomes. Second, the total number of patients in each group is small, which might lead to unreliable estimated values. Third, some results, especially 12-month mortality and VAS dyspnea scores after treatments, have obvious heterogeneity, which may influence the quality of the results. Fourth, some outcome score is low according to GRADE, which might weaken the quality of some results. Fifth, we cannot fully match the types of confounding factors (such as the size of inserted chest tubes), which may have influenced the pooled results.

## Conclusion

The TP group had comparable survival, increased pleurodesis rates, a reduced total volume of drainage, and a reduced number of all-grade AEs. However, it is worth noting that the TP group needed more additional pleural interventions and longer hospital stays than the IPC group. In addition, TP is not feasible for patients with trapped lungs. On the other hand, the IPC group needed fewer pleural interventions and shorter hospital stays than the TP group. Nonetheless, one cannot avoid the fact that the IPC group had the nuisance of a long-term in situ drain and an increased risk of infection. The latent limitations of our meta-analysis indicate that additional high quality, well-designed RCTs are needed to better determine the roles of TP and IPC in clinical situations.

## Supplementary information

**Additional file 1: Table S1** PRISMA 2009 Checklist.

**Additional file 2: Table S2** Search strategy.

**Additional file 3: Table S3**. Quality assessment of all included studies using the new version 2 of the Cochrane risk-of-bias tool.

**Additional file 4: Table S4** GRADE Quality assessment by therapeutic strategy and study design for the outcomes (survival, pleurodesis rates, further pleural procedures, symptoms, and adverse events).

**Additional file 5: Figure S1** Forest plot of 3-months mortality (A) and 12-months mortality (B) associated with TP versus IPC.

**Additional file 6: Figure S2** Forest plots of VAS dyspnea at baseline (A), VAS dyspnea scores after treatments (B), and quality of life (C) associated with TP versus IPC.

**Additional file 7: Figure S3** Sensitivity analysis of all AEs (A) and serious AEs (B) associated with TP versus IPC.

**Additional file 8: Figure S4** Begg’s and Egger’s tests for comparisons of all AEs (A) and serious AEs (B) associated with TP versus IPC.

## Data Availability

All the data used in the study can be obtained from the original articles.

## Abbreviations

IPC: Indwelling pleural catheter
TP: Talc pleurodesis
MPE: Malignant pleural effusion
RCT: Randomized controlled trail
PRISMA: Preferred Reporting Items for Systematic Review and Meta-analysis
AEs: Adverse events
RR: Risk ratios
CI: Confidence intervals
WMD: Weighted mean differences
SMD: Standardized mean differences
QoL: Quality of life
SD: Standard deviation
OR: Odds ratio
QLQ: Quality of life questionnaire
EQ5D: EuroQol 5 dimensions
VAS: Visual analog scale
GRADE: Grades of Recommendations Assessment, Development and Evaluation
