# Supplementary material for: Talc pleurodesis versus indwelling pleural catheter among patients with malignant pleural effusion: a meta-analysis of randomized controlled trials
Source: World J Surg Oncol. 2020 Jul 23;18:184. doi: 10.1186/s12957-020-01940-6 (PMC7379784; doi:10.1186/s12957-020-01940-6)
Supplement: Supplementary file 2 — Additional file 2: Table S2 Search strategy. [file 12957_2020_1940_MOESM2_ESM.docx]

**Table S2. Search strategy**

The combined terms used were: “Indwelling Pleural catheter”, “Talc pleurodesis” and “Malignant pleural effusion”.

**PubMed**

The database was searched on February 2, 2020, n= 78.

Search Strategy:

 (Indwelling pleural catheter [MeSH term]) AND (Talc pleurodesis [MeSH term]) AND (Pleural Effusion, Malignant [MeSH term] OR Effusion, Malignant Pleural [Text Word] OR Effusions, Malignant Pleural [Text Word] OR Malignant Pleural Effusion [Text Word] OR Malignant Pleural Effusions [Text Word] OR Pleural Effusions, Malignant[ Text Word])

**Web of Science**

The database was searched on February 2, 2020, n= 73.

Search Strategy:

1 TOPIC: (“ Indwelling pleural catheter”)

2 TOPIC: (“Talc pleurodesis”)

3 TOPIC: (“Pleural Effusion, Malignant” OR “Effusion, Malignant Pleural” OR “Effusions, Malignant Pleural” OR “Malignant Pleural Effusion” OR “Malignant Pleural Effusions” OR “Pleural Effusions, Malignant”)

4 #1 AND #2 AND #3 (73)

**EMBASE**

The database was searched on February 2, 2020, n= 187.

Search Strategy:

('Indwelling pleural catheter':ti,ab,kw) AND ('Talc pleurodesis':ti,ab,kw) AND ('Pleural Effusion, Malignant' OR 'Effusion, Malignant Pleural' OR 'Effusions, Malignant Pleural' OR 'Malignant Pleural Effusion' OR 'Malignant Pleural Effusions' OR 'Pleural Effusions, Malignan':ti,ab,kw)

**Cochrane Library**

The database was searched on February 2, 2020, n= 49.

Search Strategy:

(“Indwelling pleural catheter”): ti,ab,kw AND (“Talc pleurodesis”): ti,ab,kw AND (“Pleural Effusion, Malignant” OR “Effusion, Malignant Pleural” OR “Effusions, Malignant Pleural” OR “Malignant Pleural Effusion” OR “Malignant Pleural Effusions” OR “Pleural Effusions, Malignant”): ti,ab,kw - (Word variations have been searched)

**Ovid MEDLINE**

The database was searched on February 2, 2020, n= 107.

Search Strategy:

1 Indwelling pleural catheter

2 Talc pleurodesis

3 Pleural Effusion, Malignant

4 Effusion, Malignant Pleural

5 Effusions, Malignant Pleural

6 Malignant Pleural Effusion

7 Malignant Pleural Effusions

8 Pleural Effusions, Malignan

9 or/3-8 [Malignant Pleural Effusion]

10 1 and 2 and 9

5 limit 10 to humans (41)

**ScienceDirect**

The database was searched on February 2, 2020, n= 158.

Search Strategy:

Title, abstract, keywords: ((“Indwelling pleural catheter”) AND (“Talc pleurodesis”) AND (“Pleural Effusion, Malignant” OR “Effusion, Malignant Pleural” OR “Effusions, Malignant Pleural” OR “Malignant Pleural Effusion” OR “Malignant Pleural Effusions” OR “Pleural Effusions, Malignant”))

**Scopus**

The database was searched on February 2, 2020, n= 50.

Search Strategy:

TITLE-ABS-KEY ((“Indwelling pleural catheter”) AND (“Talc pleurodesis”) AND (“Pleural Effusion, Malignant” OR “Effusion, Malignant Pleural” OR “Effusions, Malignant Pleural” OR “Malignant Pleural Effusion” OR “Malignant Pleural Effusions” OR “Pleural Effusions, Malignant”)).
