# Supplementary material for: Talc pleurodesis versus indwelling pleural catheter among patients with malignant pleural effusion: a meta-analysis of randomized controlled trials
Source: World J Surg Oncol. 2020 Jul 23;18:184. doi: 10.1186/s12957-020-01940-6 (PMC7379784; doi:10.1186/s12957-020-01940-6)
Supplement: Supplementary file 3 — Additional file 3: Table S3. Quality assessment of all included studies using the new version 2 of the Cochrane risk-of-bias tool. [file 12957_2020_1940_MOESM3_ESM.docx]

**Table S3.** Quality assessment of all included studies using the new version 2 of the Cochrane risk-of-bias tool.

| **RCT** | | **Domain 1: Risk of bias arising from the randomization process** | **Domain 2: Risk of bias due to deviations from the intended interventions** | **Domain 3: Risk of bias due to missing outcome data** | **Domain 4: Risk of bias in measurement**  **of the outcome** | **Domain 5: Risk of bias in selection of the reported result** |
| --- | --- | --- | --- | --- | --- | --- |
|  |  |  |  |  |  |  |
| 2012 | Demmy, et al [20] | low-risk | some-concern | some-concern | some-concern | low-risk |
| 2012 | Davies, et al [21] | low-risk | low-risk | low-risk | low-risk | low-risk |
| 2017 | Boshuizen, et al [22] | low-risk | some-concern | low-risk | some-concern | low-risk |
| 2017 | Thomas, et al [23] | low-risk | some-concern | low-risk | low-risk | some-concern |

**Abbreviations:** RCT: randomized controlled trial.
