# Supplementary material for: Talc pleurodesis versus indwelling pleural catheter among patients with malignant pleural effusion: a meta-analysis of randomized controlled trials
Source: World J Surg Oncol. 2020 Jul 23;18:184. doi: 10.1186/s12957-020-01940-6 (PMC7379784; doi:10.1186/s12957-020-01940-6)
Supplement: Supplementary file 4 — Additional file 4: Table S4 GRADE Quality assessment by therapeutic strategy and study design for the outcomes (survival, pleurodesis rates, further pleural procedures, symptoms, and adverse events). [file 12957_2020_1940_MOESM4_ESM.docx]

**Table S4** GRADE Quality assessment by therapeutic strategy and study design for the outcomes (survival, pleurodesis rates, symptoms, further pleural procedures, and complications).

| **Primary outcomes** | **No. of Studies** | **No. of participants** | | **Differences ^a^（95%CI）** | **Quality assessment** | | | | | **Quality** | |
| --- | --- | --- | --- | --- | --- | --- | --- | --- | --- | --- | --- |
|  |  | **TP** | **IPC** |  | **Risk of bias^b^** | **Inconsistency** | **Indirectness** | **Imprecision** | **Publication bias ^c^** |  |  |
| **Survival** |  |  |  |  |  |  |  |  |  |  | |
| 3-months mortality | 2 | 24/83 | 25/80 | 0.80 [0.29, 2.16] | No | Serious (-1) | No indirectness | No imprecision | Unlikely | medium | |
| 12-months mortality | 2 | 98/126 | 93/126 | 1.03 [0.76, 1.40] | No | Serious (-1) | No indirectness | No imprecision | Unlikely | medium | |
| **Pleurodesis** |  |  |  |  |  |  |  |  |  |  | |
| Pleurodesis rates | 2 | 73/83 | 44/78 | 1.56 [1.26, 1.92] | No | No inconsistency | No indirectness | No imprecision | Unlikely | high | |
| **Pleural interventions** |  |  |  |  |  |  |  |  |  |  | |
| Further pleural interventions | 3 | 43/174 | 13/172 | 3.24 [1.82, 5.78] | No | No inconsistency | No indirectness | No imprecision | Unlikely | high | |
| **Symptoms** |  |  |  |  |  |  |  |  |  |  | |
| VAS dyspnea scores at |  |  |  |  | No | No inconsistency | No indirectness | No imprecision | Unlikely | high | |
| baseline (mm) | 2 | 126/126 | 126/126 | -2.32 [-8.59, 3.94] |  |  |  |  |  |  |  |
| VAS dyspnea scores after treatments (mm) |  |  |  |  | No | Very serious (-2) | No indirectness | No imprecision | Unlikely | low | |
|  | 2 | 125/125 | 125/125 | 0.93 [-1.79, 3.65] |  |  |  |  |  |  |  |
| **Quality of life** |  |  |  |  |  |  |  |  |  |  | |
| Quality of life | 2 | 127/127 | 123/123 | -1.50 [-3.80, 0.80] | No | Serious (-1) | No indirectness | No imprecision | Unlikely | medium | |
| **Complications** |  |  |  |  |  |  |  |  |  |  | |
| All AEs | 3 | 75/173 | 94/171 | 0.67 [0.29, 1.54] | No | Very serious (-2) | No indirectness | No imprecision | Unlikely | low | |
| Serious AEs | 3 | 11/154 | 17/153 | 0.63 [0.31, 1.28] | No | Serious (-1) | No indirectness | No imprecision | Unlikely | medium | |
| Serious pleural infection | 2 | 2/126 | 7/126 | 0.28 [0.06, 1.32] | No | No inconsistency | No indirectness | No imprecision | Unlikely | high | |
| Dyspnea/Breathlessness | 2 | 8/119 | 7/119 | 1.15 [0.43, 3.06] | No | No inconsistency | No indirectness | No imprecision | Unlikely | high | |
| Serious pain | 2 | 1/100 | 3/101 | 0.61 [0.03, 12.92] | No | Serious (-1) | No indirectness | No imprecision | Unlikely | medium | |
| Catheter blockage | 2 | 1/126 | 13/126 | 0.11 [0.02, 0.57] | No | No inconsistency | No indirectness | No imprecision | Unlikely | high | |
| Cellulitis | 2 | 1/126 | 10/126 | 0.14 [0.03, 0.77] | No | No inconsistency | No indirectness | No imprecision | Unlikely | high |  |

**Abbreviations:** IPC: indwelling pleural catheter; TP: talc pleurodesis; CI: confidence interval; AEs: adverse effects; CI: confidence interval.

^a^ Differences: risk ratio (RR) for 3-months mortality; 12-months mortality; pleurodesis rates; further pleural interventions and complications. Weighted mean differences (WMD) for baseline VAS dyspnea scores (mm) and VAS dyspnea scores after therapy (mm), and standardized mean differences (SMD) for quality of life.

^b^ Risk of bias of all included trials are assessed by the new version 2 of the Cochrane risk-of-bias tool.

^c^ Publication bias was assessed by Egger’s and Begg’s tests.
