# Supplementary figures and images for: Talc pleurodesis versus indwelling pleural catheter among patients with malignant pleural effusion: a meta-analysis of randomized controlled trials
Source: World J Surg Oncol. 2020 Jul 23;18:184. doi: 10.1186/s12957-020-01940-6 (PMC7379784; doi:10.1186/s12957-020-01940-6)

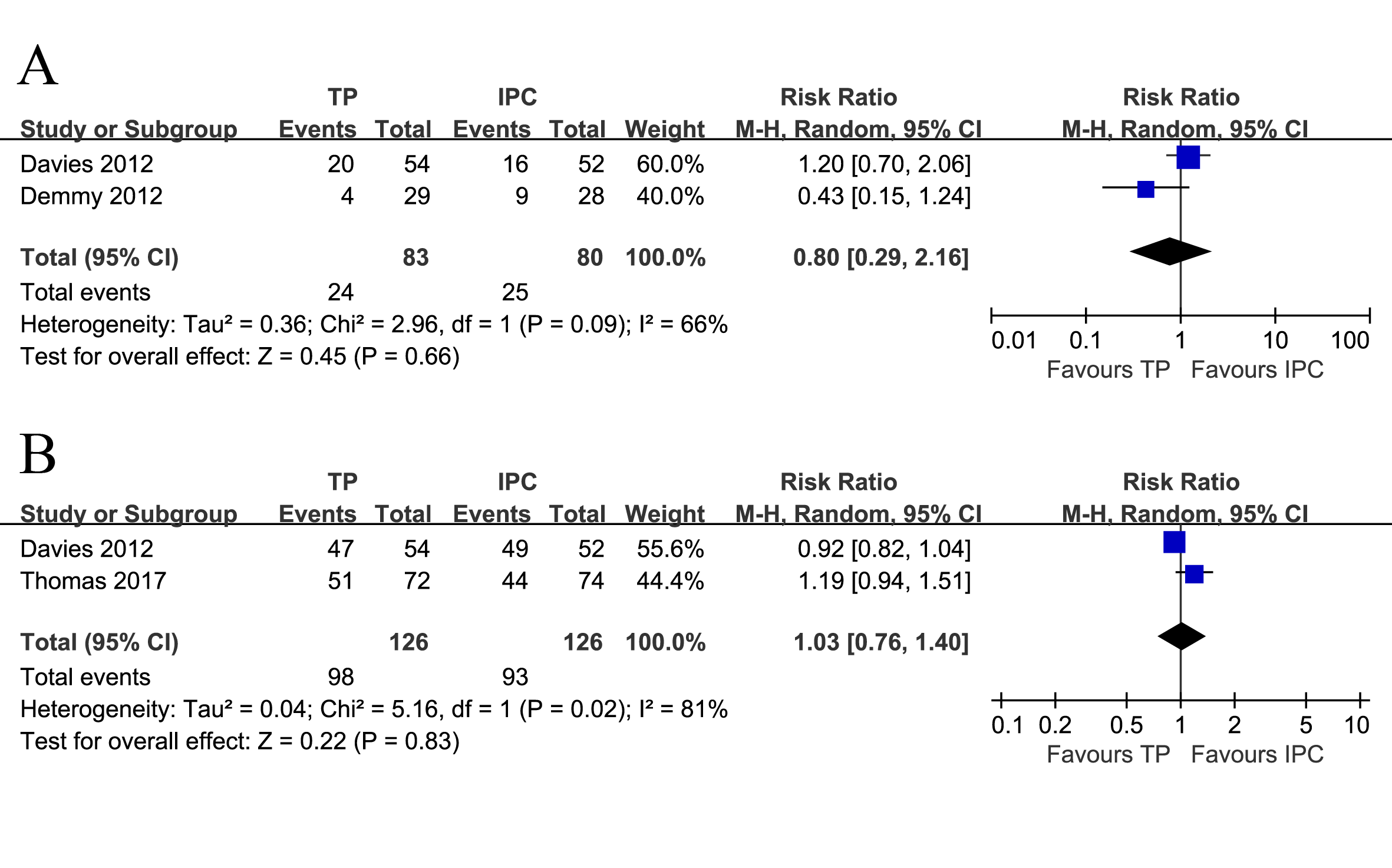

Supplement: Supplementary file 5 — Additional file 5: Figure S1 Forest plot of 3-months mortality (A) and 12-months mortality (B) associated with TP versus IPC. [file 12957_2020_1940_MOESM5_ESM.tif]

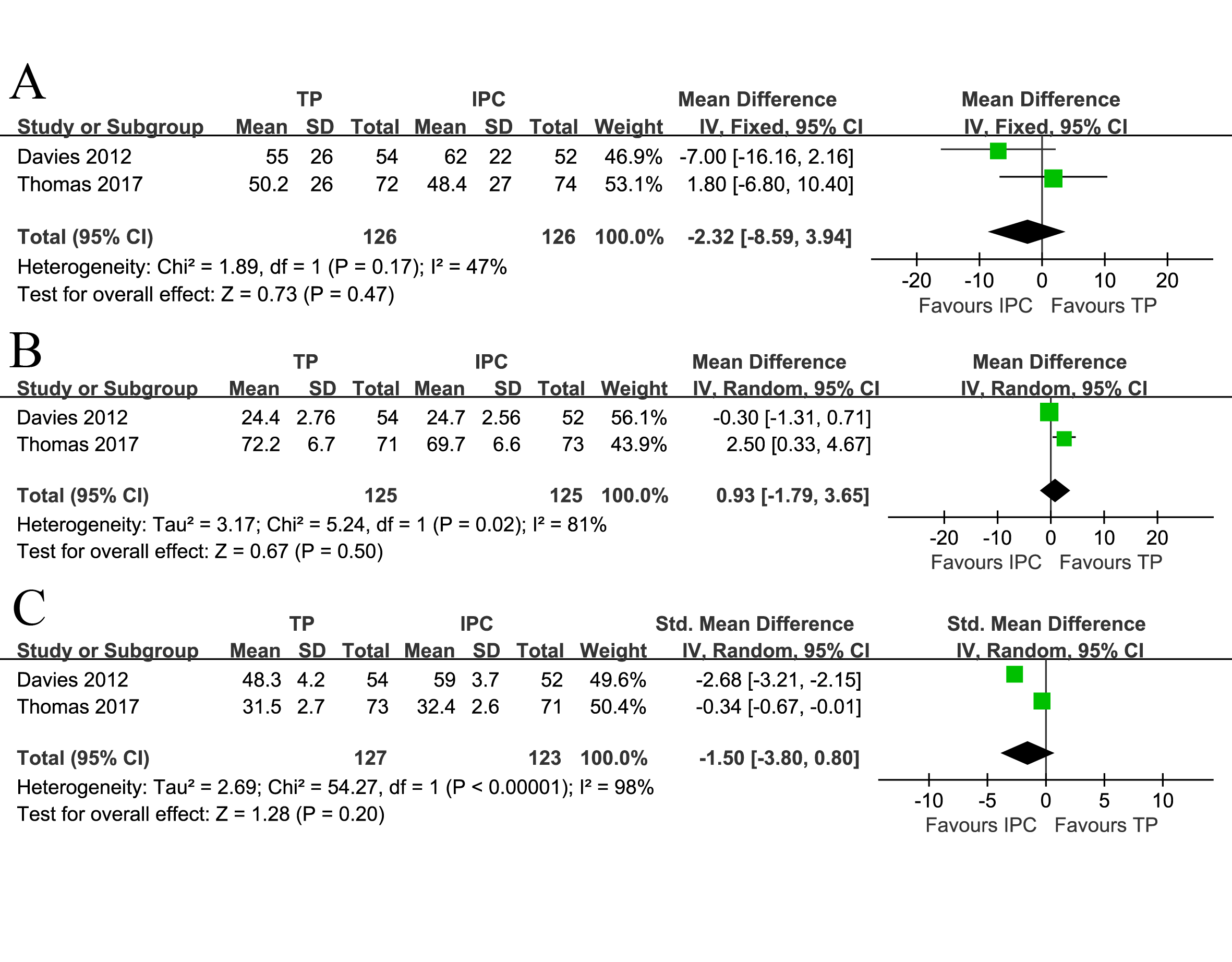

Supplement: Supplementary file 6 — Additional file 6: Figure S2 Forest plots of VAS dyspnea at baseline (A), VAS dyspnea scores after treatments (B), and quality of life (C) associated with TP versus IPC. [file 12957_2020_1940_MOESM6_ESM.tif]

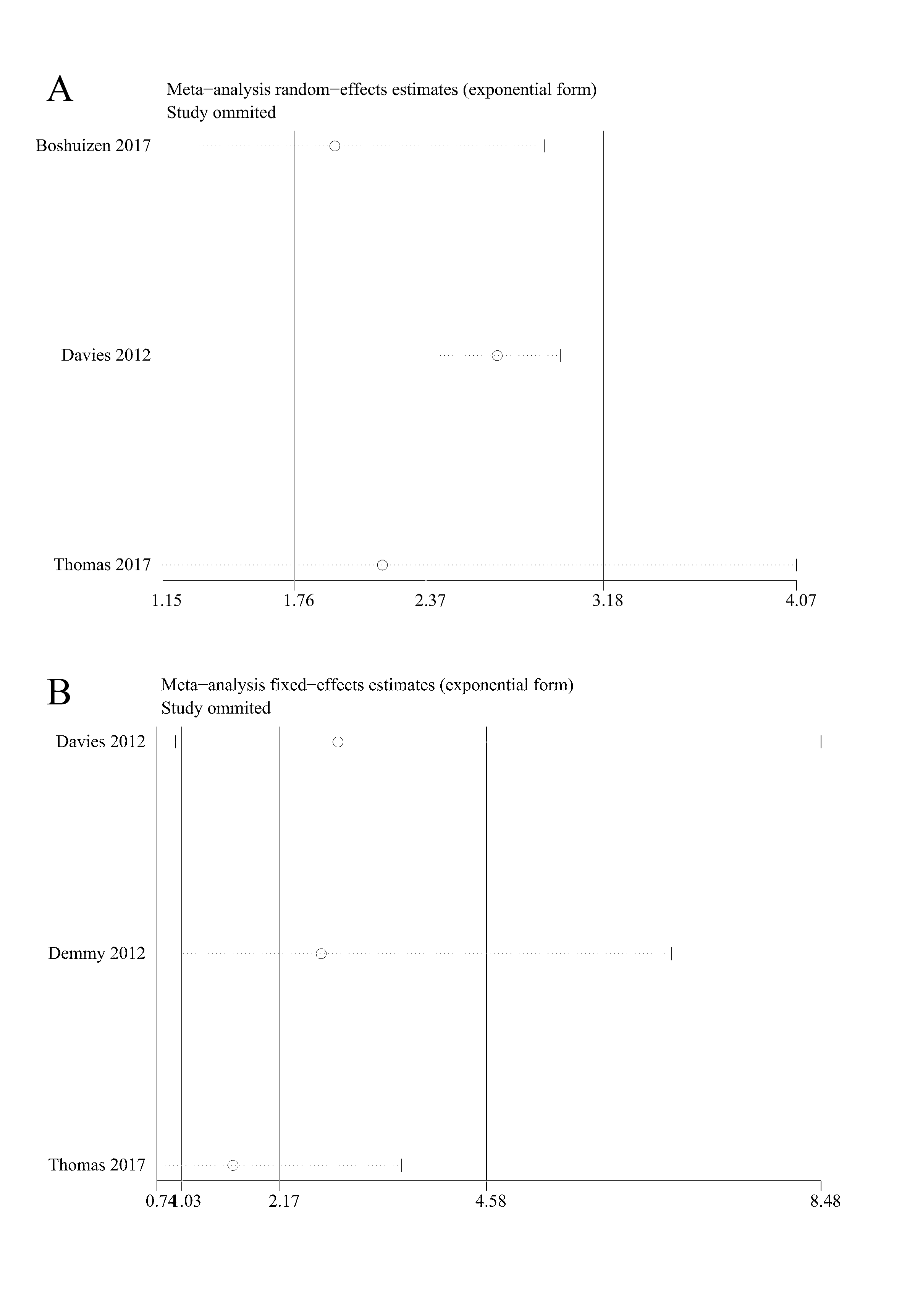

Supplement: Supplementary file 7 — Additional file 7: Figure S3 Sensitivity analysis of all AEs (A) and serious AEs (B) associated with TP versus IPC. [file 12957_2020_1940_MOESM7_ESM.tif]

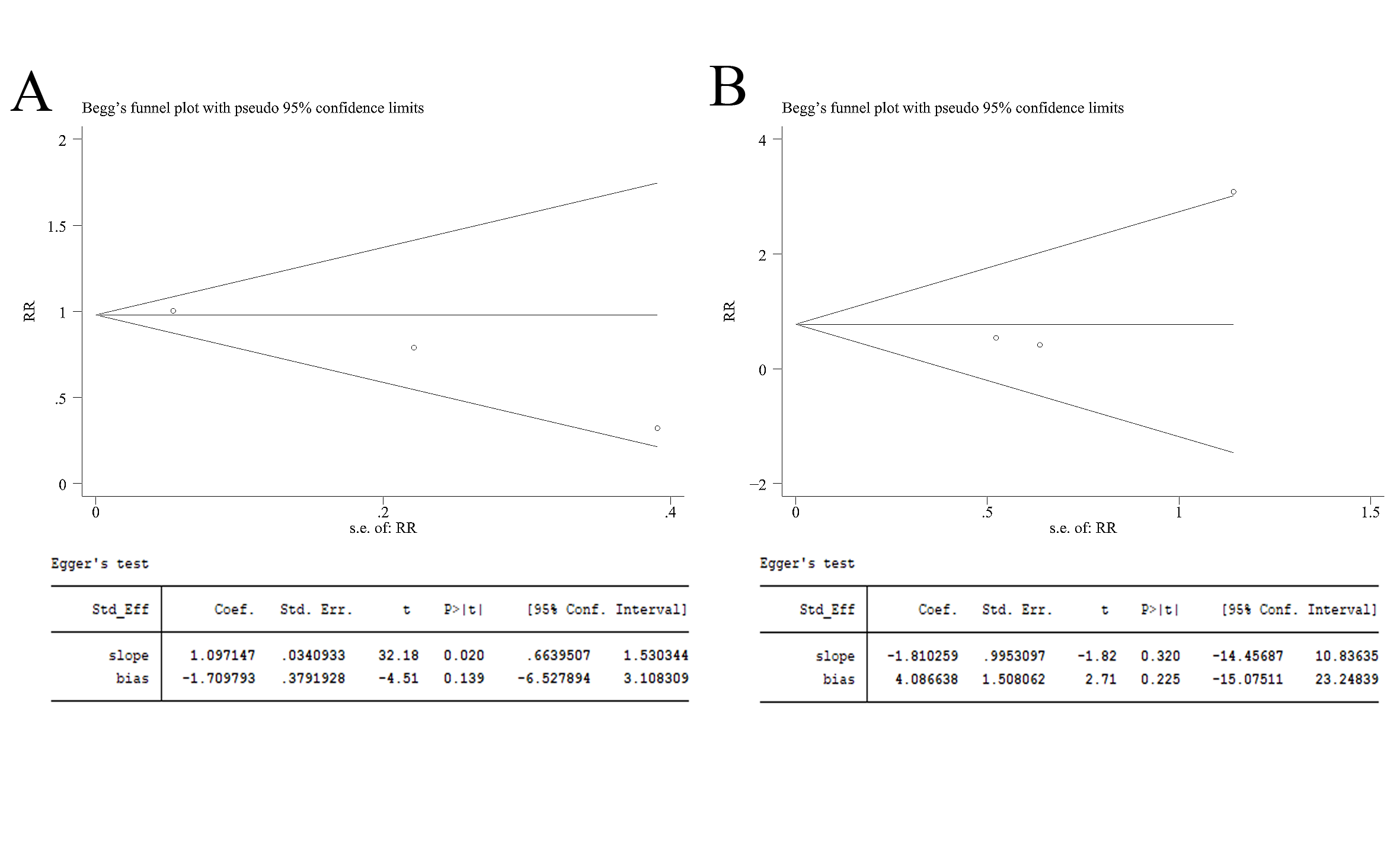

Supplement: Supplementary file 8 — Additional file 8: Figure S4 Begg’s and Egger’s tests for comparisons of all AEs (A) and serious AEs (B) associated with TP versus IPC. [file 12957_2020_1940_MOESM8_ESM.tif]
